# Successful Conservative Management of Emphysematous Cystitis Following COVID-19 Infection

**DOI:** 10.7759/cureus.108844

**Published:** 2026-05-14

**Authors:** Ina Bahl, Amit Agrawal, Neeraj Nagarwal, Surendra Saini, Ram Sevak

**Affiliations:** 1 Surgery, All India Institute of Medical Sciences, New Delhi, New Delhi, IND; 2 Urology, Command Hospital, Chandigarh, IND; 3 Neurosurgery, Sawai Man Singh (SMS) Medical College, Jaipur, IND; 4 Neurosurgery, Jhalawar Medical College, Jhalawar, IND

**Keywords:** antibiotics, computed tomography, covid, diabetes, emphysematous cystitis

## Abstract

Emphysematous cystitis (EC) is a rare, potentially life-threatening urinary tract infection characterized by gas within the bladder wall or lumen due to gas-forming organisms. It most commonly occurs in patients with uncontrolled diabetes mellitus. Reports following COVID-19 remain limited.

A 56-year-old male presented five weeks after recovery from COVID-19 pneumonia with fever, flank pain, and lower abdominal pain. Investigations revealed severe hyperglycemia, leukocytosis, and pyuria. CT of the kidneys, ureters, and bladder demonstrated intramural gas with anterior bladder wall dehiscence. Urine culture grew *Enterococcus faecalis*. The patient was managed conservatively with bladder drainage, glycemic control, and prolonged culture-directed antibiotics. Despite extensive disease, he remained clinically stable and showed complete recovery on follow-up imaging.

This case highlights that conservative management can be successful even in extensive EC with bladder wall involvement in carefully selected, stable patients. The temporal association with COVID-19 is noted, but causality cannot be established.

## Introduction

Emphysematous cystitis (EC) is a rare form of complicated UTI characterized by the presence of gas within the bladder wall or lumen, produced by gas-forming microorganisms [1,2]. The condition occurs most commonly in individuals with impaired host defenses, with diabetes mellitus representing the most frequently reported risk factor [1,2]. Additional predisposing factors include urinary stasis, bladder outlet obstruction, neurogenic bladder, indwelling urinary catheters, and immunosuppressive therapies [2,3].

Clinical manifestations of EC vary widely and may range from mild lower urinary tract symptoms to severe sepsis. Patients may present with abdominal pain, dysuria, hematuria, pneumaturia, or systemic symptoms such as fever and malaise. Early recognition of the condition is essential because delayed diagnosis can lead to complications, including bladder wall necrosis, bladder rupture, and death [1,2].

CT imaging plays a crucial role in the diagnosis of emphysematous UTIs because it allows accurate visualization of intramural gas, assessment of disease extent, and identification of associated complications [4]. Prompt diagnosis enables early initiation of bladder drainage, antimicrobial therapy, and correction of underlying metabolic abnormalities, which are key components of successful treatment.

The association between EC and COVID-19 has only rarely been reported. SARS-CoV-2 may predispose patients to secondary infections through endothelial injury, immune dysregulation, and metabolic disturbances [4]. Viral entry into host cells occurs through angiotensin-converting enzyme 2 receptors, which are expressed in multiple organs, including the bladder urothelium, suggesting that the urinary tract may be susceptible to viral-mediated injury [5].

In addition, corticosteroid therapy, frequently administered during COVID-19 treatment, may exacerbate hyperglycemia or unmask previously undiagnosed diabetes, thereby increasing susceptibility to severe UTIs, including EC [4]. Post-COVID inflammatory changes have also been associated with worsening lower urinary tract symptoms in some patients [6].

We report a case of EC occurring following COVID-19 infection in a patient with uncontrolled diabetes mellitus, with extensive anterior bladder wall dehiscence that was successfully managed with conservative therapy.

The aim of this report is to highlight the successful conservative management of EC with bladder wall dehiscence and to emphasize the importance of early diagnosis and appropriate patient selection.

## Case presentation

A 56-year-old male presented to the ED five weeks after recovery from COVID-19 pneumonia with a two-week history of right flank pain and lower abdominal pain associated with high-grade fever and chills. The patient reported progressive worsening of abdominal discomfort along with generalized weakness and malaise. He denied hematuria or pneumaturia at presentation. His past medical history was notable for poorly controlled diabetes mellitus, diagnosed during the COVID-19 illness.

On clinical examination, the patient appeared febrile and ill-looking. His body temperature was 102°F. Abdominal examination revealed suprapubic tenderness with localized guarding. Right renal angle tenderness was present. Digital rectal examination did not demonstrate prostatomegaly or findings suggestive of prostatitis. Based on the clinical presentation, the patient was initially admitted with a provisional diagnosis of acute pyelonephritis.

Initial laboratory investigations demonstrated significant leukocytosis, severe hyperglycemia, and abnormal urine microscopy suggestive of UTI. The laboratory parameters, along with their reference ranges, are summarized in Table 1.

**Table 1:** Laboratory parameters at admission and at final follow-up.

Investigation	On admission	At final follow-up	Reference range
Random blood glucose	490 mg/dL	130 mg/dL	70-140 mg/dL
HbA1c	8.00%	6.50%	<5.7%
Total leukocyte count	24,000/mm³	9,500/mm³	4,000-11,000/mm³
Neutrophils	88%	65%	40-75%
Urine microscopy	10-16 RBCs/HPF, numerous pus cells	1-2 RBCs/HPF	<5 RBCs/HPF
Urine protein	1+	Negative	Negative
Serum creatinine	1.0 mg/dL	0.9 mg/dL	0.6-1.2 mg/dL

These findings were consistent with a severe UTI occurring in the background of uncontrolled diabetes mellitus.

Given the severity of symptoms and laboratory findings, urgent non-contrast CT of the kidney, ureter, and bladder (CT KUB) was performed. Imaging demonstrated multiple intramural gas foci within the bladder wall, associated with focal anterior bladder wall dehiscence and surrounding perivesical fat stranding. These radiological findings were diagnostic of EC.

The CT scan also demonstrated right ureteric wall thickening; however, no radiological evidence of acute pyelonephritis was identified. CT imaging remains the most reliable modality for diagnosing emphysematous UTIs because it allows precise identification of gas within the bladder wall and assessment of the extent of disease [7].

**Figure 1:**
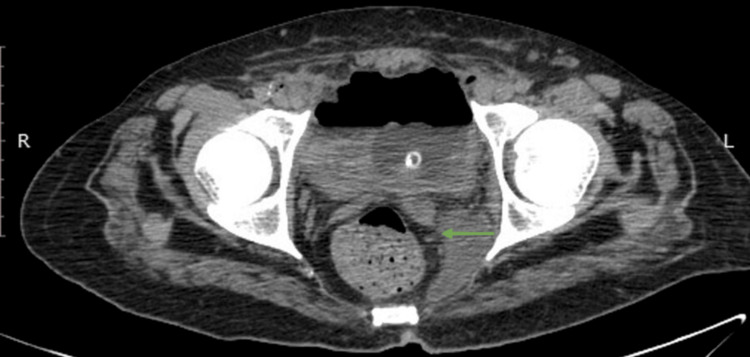
Non-contrast CT KUB showing multiple intramural gas foci within the bladder wall (arrow), with focal anterior bladder wall dehiscence and surrounding perivesical fat stranding. CT KUB: Computed tomography of the kidney, ureter, and bladder.

The initial CT findings are illustrated in Figure 1, which demonstrates intramural gas within the bladder wall and focal disruption of the anterior bladder wall.

A urethral Foley catheter was inserted immediately, resulting in drainage of turbid urine containing debris. The patient was initiated on insulin therapy to achieve strict glycemic control, along with empirical broad-spectrum intravenous antibiotics. Urine culture subsequently demonstrated growth of *Enterococcus faecalis*, following which antibiotic therapy was modified according to culture sensitivity results.

The presence of anterior bladder wall dehiscence raised concern regarding possible bladder rupture. Surgical intervention, including bladder wall debridement, was initially considered. However, imaging suggested that the rupture was contained, and the patient remained hemodynamically stable with preserved renal function and no evidence of peritonitis. Considering the potential morbidity associated with extensive bladder surgery and the possibility of requiring reconstructive procedures, conservative management was pursued with close clinical monitoring.

During hospitalization, repeated catheter obstruction occurred due to marked pyuria and debris formation. To address this problem, a three-way Foley catheter was inserted, and continuous bladder irrigation was initiated to prevent catheter blockage and facilitate bladder drainage.

**Figure 2:**
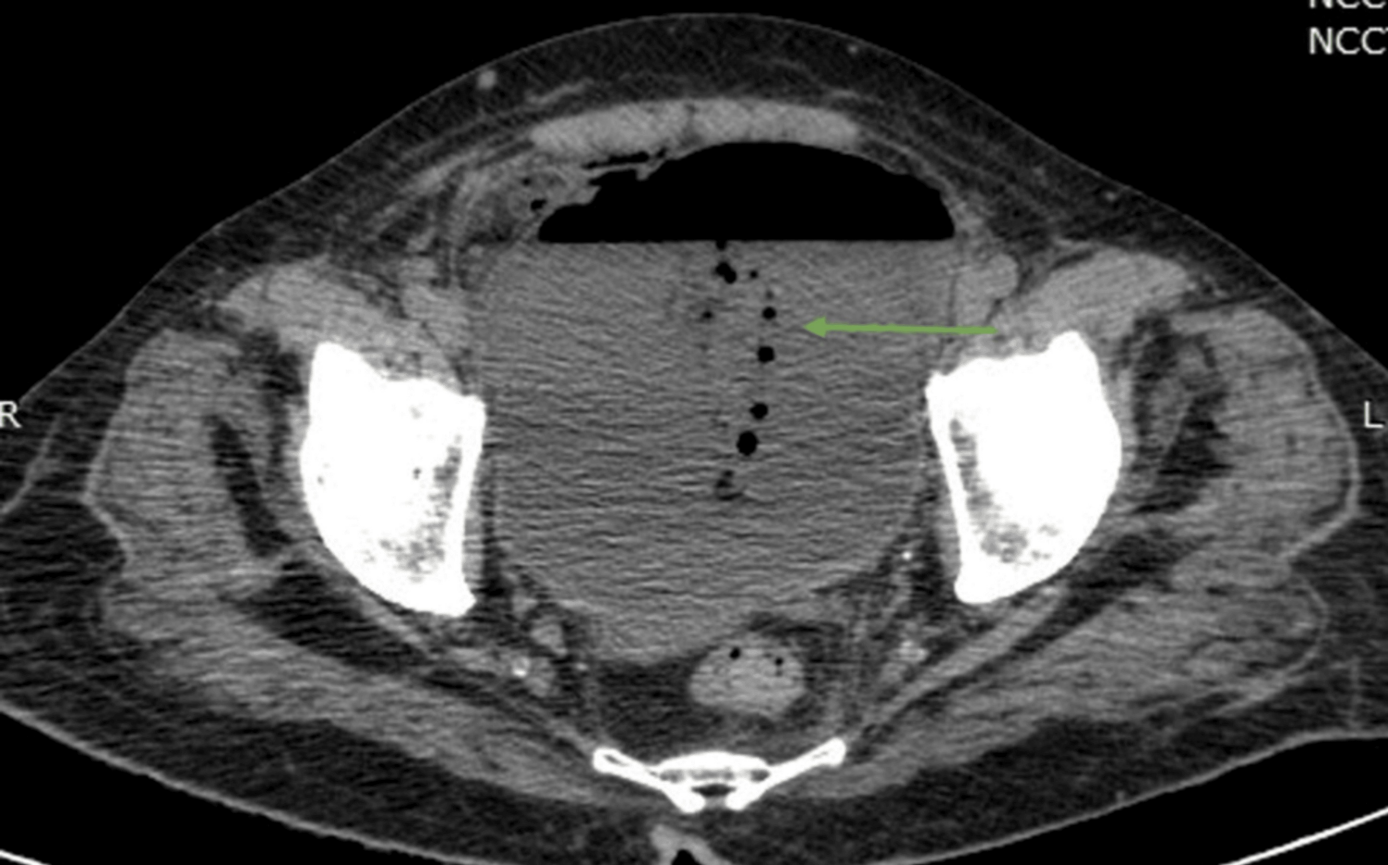
Follow-up CT KUB performed at four weeks, demonstrating persistent intramural gas within the bladder wall and minimal reduction in the perivesical inflammatory collection (arrow). CT KUB: Computed tomography of the kidney, ureter, and bladder.

Although the patient remained clinically stable, persistent leukocytosis and turbid urine suggested ongoing infection. Repeat CT imaging performed four weeks after admission demonstrated persistent intramural gas and minimal reduction in the perivesical inflammatory collection. These findings are shown in Figure 2.

Antibiotic therapy was continued for a total duration of eight weeks, initially administered intravenously and subsequently transitioned to oral therapy once clinical improvement was observed.

**Figure 3:**
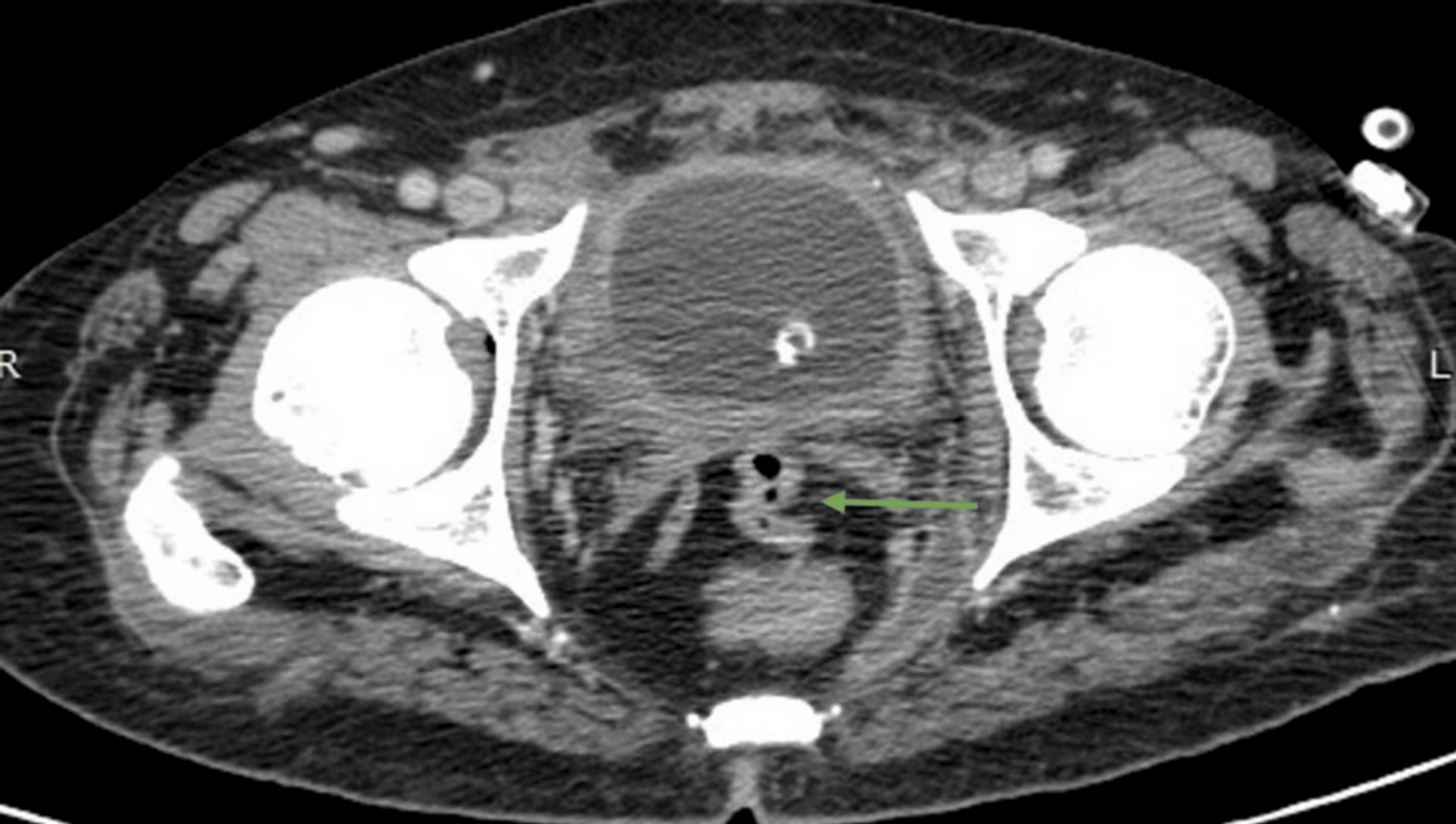
Contrast-enhanced CT KUB performed at eight weeks, demonstrating significant reduction in intramural gas and healing of the bladder wall, with a small residual anterosuperior bladder wall dehiscence (arrow). CT KUB: Computed tomography of the kidney, ureter, and bladder.

At eight weeks of treatment, laboratory parameters showed improvement. The leukocyte count decreased to below 12,000/mm³ (reference range 4,000-11,000/mm³), indicating resolution of systemic infection. Repeat contrast-enhanced CT imaging demonstrated marked reduction of intramural gas, with progressive healing of the bladder wall and only a small residual anterosuperior bladder wall defect. These findings are illustrated in Figure 3.

After 60 days of hospitalization, the patient was discharged with an indwelling urinary catheter. The catheter was removed four weeks later during follow-up after confirmation of adequate bladder healing. At the final follow-up visit, the patient reported normal voiding without dysuria or abdominal pain, and laboratory parameters had returned to normal values (Table 1).

The clinical course followed a clear chronological progression. The patient developed symptoms approximately two weeks prior to presentation and was admitted five weeks after recovery from COVID-19. A CT scan performed at admission confirmed the diagnosis of EC. Despite initial conservative management, follow-up imaging at four weeks demonstrated persistent intramural gas with minimal improvement. Given the patient’s stable clinical status, conservative therapy was continued. Subsequent imaging at eight weeks showed significant resolution of intramural gas and progressive bladder wall healing, following which the patient was discharged and later underwent successful catheter removal at follow-up.

## Discussion

EC is an uncommon but clinically significant form of complicated UTI characterized by the presence of gas within the bladder wall or lumen, produced by gas-forming microorganisms. Although the condition is rare, it can progress to severe sepsis, bladder wall necrosis, or rupture if not diagnosed promptly. In a large review of 135 reported cases by Thomas AA et al., the overall mortality rate associated with EC was approximately 7%, highlighting the potentially serious nature of the disease [1].

Diabetes mellitus is the most important predisposing factor for EC. Hyperglycemia promotes bacterial fermentation of glucose within tissues, resulting in carbon dioxide and hydrogen gas production. In addition, diabetic microvascular disease may impair tissue perfusion and immune response, thereby facilitating bacterial proliferation within the bladder wall. Previous studies have reported that nearly two-thirds of patients diagnosed with EC have underlying diabetes mellitus [1,2]. In the present case, the patient demonstrated severe hyperglycemia, with a random blood glucose level of 490 mg/dL and an elevated hemoglobin A1c (HbA1c) of 8%, suggesting poorly controlled diabetes that likely contributed to disease development.

Other recognized risk factors for EC include urinary tract obstruction, neurogenic bladder, chronic urinary catheterization, and immunosuppressive states [2,3]. Urinary stasis in such conditions provides a favorable environment for bacterial growth and gas formation. The microbiological profile of EC typically includes gas-forming organisms such as *Escherichia coli*, *Klebsiella pneumoniae*, and *Enterococcus* species [1,2,7]. In our patient, urine culture grew *Enterococcus faecalis*, which has been reported as a less common but recognized pathogen in emphysematous UTIs.

An unusual aspect of this case was the recent history of COVID-19 infection. Although EC has not been widely reported in association with COVID-19, several biological mechanisms may explain this relationship. SARS-CoV-2 infection has been shown to cause endothelial injury and systemic inflammatory responses that predispose patients to secondary infections [4]. Viral entry occurs through angiotensin-converting enzyme 2 (ACE2) receptors, which are expressed in multiple organs, including the bladder urothelium [5]. The presence of ACE2 receptors within the urinary tract suggests that viral infection may directly or indirectly affect bladder physiology and immune defense mechanisms.

In addition, treatment strategies employed during COVID-19 illness may contribute to metabolic and immunological disturbances. Corticosteroids are frequently used to manage severe COVID-19 pneumonia and may exacerbate hyperglycemia or precipitate previously undiagnosed diabetes mellitus. Hyperglycemia, in turn, creates an environment favorable for gas-forming bacterial infections. Post-COVID inflammatory changes have also been associated with worsening lower urinary tract symptoms in some patients, further suggesting that the urinary system may be affected during or after SARS-CoV-2 infection [6].

CT imaging is considered the diagnostic modality of choice for EC. CT allows accurate visualization of intramural gas within the bladder wall and provides valuable information regarding disease extent and potential complications such as bladder rupture or perivesical abscess formation [7]. In the present case, CT imaging demonstrated extensive intramural gas with anterior bladder wall dehiscence and perivesical fat stranding. Despite the severity of these findings, the rupture appeared contained, allowing consideration of conservative management.

Standard management of EC typically includes bladder drainage, broad-spectrum antimicrobial therapy, strict glycemic control, and correction of underlying predisposing factors [1,2,7]. Surgical intervention is generally reserved for patients who develop complications such as bladder necrosis, uncontrolled sepsis, or extensive bladder wall destruction [2,8]. In the current case, surgical debridement was initially considered because of the presence of anterior bladder wall dehiscence. However, the patient remained hemodynamically stable, and imaging suggested a contained rupture without evidence of generalized peritonitis. Consequently, conservative management was chosen.

Several factors likely contributed to the favorable outcome observed in this patient. Continuous bladder drainage prevented urinary stasis and facilitated removal of infected urine. Strict glycemic control reduced the metabolic substrate available for bacterial fermentation. Prolonged culture-directed antibiotic therapy ensured adequate eradication of the infecting organism. Close clinical monitoring allowed early detection of complications that might have required surgical intervention.

Although extensive bladder wall involvement is often considered an indication for surgery, this case demonstrates that carefully selected patients with stable clinical parameters may achieve successful outcomes with conservative therapy. Similar observations have been reported in previous studies, where aggressive bladder drainage and antimicrobial therapy resulted in complete resolution of infection without surgical intervention [1,2].

Although a temporal association with COVID-19 infection was observed in this case, a causal relationship cannot be established. Uncontrolled diabetes remains the most significant contributing factor. This case emphasizes the importance of early CT diagnosis, appropriate antibiotic therapy, and careful patient selection for conservative management, even in extensive disease.

Limitations

This report represents a single case, limiting generalizability. The temporal association with COVID-19 does not establish causation. Further studies are required to explore any potential relationship.

## Conclusions

EC is a rare but serious infection that requires early diagnosis and prompt management. CT imaging plays a critical role in diagnosis and follow-up. Conservative management can be effective, even in extensive disease, in clinically stable patients. Any association with COVID-19 remains hypothesis-generating and requires further investigation.
